# Epinephrine in Cardiac Arrest: Identifying a Potential Limit for Resuscitation

**DOI:** 10.5811/westjem.60840

**Published:** 2023-11-08

**Authors:** Zachary Boivin, Kevin M. Duignan, Donias Doko, Nicholas Pugliese, Trent She

**Affiliations:** * University of Connecticut, Emergency Medicine Residency, Farmington, Connecticut; † Cooper University Hospital, Department of Pharmacy, Camden, New Jersey; ‡ Hartford Hospital, Department of Emergency Medicine, Hartford, Connecticut

## Abstract

**Introduction:**

Epinephrine continues to be a fundamental part of the Advanced Cardiac Life Support algorithm despite a lack of evidence that it improves neurologically intact survival. Our aim was both to identify a potential upper limit of epinephrine use in resuscitations and to demonstrate real-world epinephrine use in different patient subgroups.

**Methods:**

This was a single-center, retrospective cohort study, conducted between August 1, 2016–July 1, 2021, of patients with medical cardiac arrest who were administered a known number of epinephrine doses. The primary outcome was neurologically intact discharge defined by a modified Rankin scale ≤3, with secondary outcomes of comparing epinephrine doses by age, rhythm, and emergency medical services vs emergency department administration of epinephrine.

**Results:**

The study included 1,330 patients, with 184 patients (13.8%) surviving to neurologically intact discharge. The primary outcome of neurologically intact discharge was found in 89 (65.4%) patients in the zero epinephrine dose group, 75 (20.0%) in the 1-3 dose group, 15 (4.3%) in the 4-6 dose group, and one (0.002%) in the ≥7 dose group (*P* < 0.001). Patients received similar amounts of epinephrine when stratified by age, while patients with shockable rhythms received more epinephrine than patients with non-shockable rhythms.

**Conclusion:**

There was a significant decrease in neurologically intact discharge with increasing number of epinephrine doses, and our data suggests that seven or more doses of epinephrine is almost always futile. While further prospective studies are needed, clinicians should consider epinephrine doses when weighing the futility or benefit of continued resuscitation efforts.

Population Health Research Capsule What do we already know about this issue?

*Epinephrine in cardiac arrest has an unclear survival benefit, and prior studies have shown increasing doses to be associated with increased mortality.*

What was the research question?

*Is there a point in resuscitation where more epinephrine doses are potentially futile?*

What was the major quantitative finding of the study?

*Neurologically intact discharge ranged from 65.4% with 0 epinephrine doses to 0.002% in the ≥7 dose group (P < 0.001).*

How does this improve population health?

*Understanding epinephrine and survival rates of cardiac arrest is important to improve outcomes and decrease emergency department resource use.*

## INTRODUCTION

Advanced Cardiovascular Life Support (ACLS) has been used for patients in cardiac arrest in both the prehospital and hospital setting following its introduction in 1974.^1^ Since that time, the guidelines have undergone several changes. Medications such as bicarbonate and calcium have lost favor,^2^ ^,^ ^3^ while epinephrine remains a mainstay in both shockable (pulseless ventricular tachycardia and ventricular fibrillation) and non-shockable (asystole and pulseless electrical activity) rhythms. The most recent ACLS guidelines recommend epinephrine use in all cardiac arrest patients as a 1 mg dose given every 3–5 minutes, along with high-quality cardiopulmonary resuscitation (CPR), until the patient has return of spontaneous circulation (ROSC).^1^ Current research, however, shows epinephrine may improve the rates of ROSC but does not improve rates of survival to hospital discharge or survival with a favorable neurologic outcome.^4^ ^–^ ^7^

The European Resuscitation Council recommends terminating resuscitative efforts after 20 minutes in the absence of reversible causes, given the unlikely event of a positive outcome.^8^ The American Heart Association recommends cessation of out-of-hospital cardiac arrest resuscitation in patients with unwitnessed, non-shockable rhythms, who do not get ROSC prior to transport.^9^

While previous studies have shown increasing epinephrine doses are associated with worse resuscitation outcomes, the exact dose of epinephrine from which there would be no further benefit is unclear.^6^ ^,^ ^10^ ^–^ ^12^ It is also unclear how age may play a role in the outcome of resuscitation when taking into account increasing epinephrine dosing. Although epinephrine is a potent inotrope and vasopressor, it is also thought to increase myocardial oxygen consumption as well as increase the risk of arrhythmias during repeated dosing. Often, patients in cardiac arrest with shockable rhythms refractory to management may actually benefit from less epinephrine, to reduce myocardial oxygen demand and to limit adrenergic stimulation, which decreases the risk for dysrhythmia.^13^ ^–^ ^17^ There are, however, no established guidelines regarding when epinephrine dosing should cease during resuscitation, the risk or benefit to neurologically intact ROSC with epinephrine use in shockable rhythms, or the total number of epinephrine doses that would be most beneficial to patients.^18^ ^,^ ^19^

We set out to conduct a study to determine a point in the resuscitation where further epinephrine dosing is potentially futile. We designed our patient groups and secondary outcomes in an a priori fashion to explore the use of epinephrine by clinicians based on patient age, cardiac rhythm, and location of cardiac arrest. These outcomes provide novel data for this controversial medication. Our a priori hypothesis was that increasing doses of epinephrine are associated with increasing mortality and worse neurologic outcomes. Additionally, we attempted to identify a potential limit of epinephrine use in cardiac arrest within our institution and to describe the use of epinephrine in the emergency department (ED) for various patient populations.

## METHODS

This was a single-center, retrospective cohort study at an academic, Level I trauma and tertiary referral center that sees greater than 100,000 ED visits per year. The study received institutional review board approval. After a joint discussion with all study members, and a one-hour training session, we manually reviewed electronic health records (EHR) for all cardiac arrest patients from August 2016, which marked the institution of our current EHR system, until July 2021. All reviewers were blinded to the study question. Patients were identified using International Classification of Diseases, 10^th^ Revision (ICD-10) codes for cardiac arrest (I46, cardiac arrest; I46.2, cardiac arrest due to underlying condition; I46.9, cardiac arrest, cause unspecified; I49.01 ventricular fibrillation; J98.9 cardiac arrest due to respiratory disorder).

**Figure 1.**
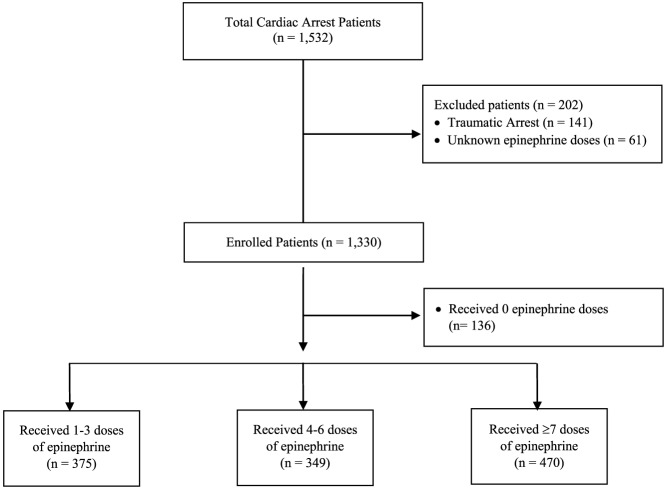
Flow diagram of study enrollment.

Inclusion criteria were patients ≥18 years old who presented to the hospital in cardiac arrest, had a cardiac arrest in the ED, or were transferred from an outside hospital after a cardiac arrest. Patients were excluded (Figure 1) if they y had a cardiac arrest only after hospital admission, a traumatic cardiac arrest, an advanced directive invoked during resuscitation, or an unknown number of epinephrine doses. Patients were also excluded if we could not determine the actual number of epinephrine doses administered based on chart review.

### Definitions

Cardiac arrest was defined as loss of pulses requiring either CPR or a shock delivered by an external defibrillator. We considered ventricular fibrillation or pulseless ventricular tachycardia as shockable rhythms, whereas asystole and pulseless electrical activity were non-shockable rhythms, per ACLS guidelines.^20^ Our department’s cardiac arrest workflow assigns one ED nurse solely to documentation during every cardiac arrest resuscitation. The nursing documentation was, therefore, the most accurate way to track medication administration, and we used this method to count epinephrine doses. For number of epinephrine doses given by emergency medical services (EMS), we also used the nursing code documentation, which started with a summary of care given by EMS of the patient presentation and transport, including any medications and interventions provided. We unfortunately did not have consistent prehospital records and so opted not to reference these due to large gaps in data collected. A patient was considered to have died in the ED if he or she was pronounced while in the ED without an admission history and physical documented.

We chose to study substance use as a comorbidity and defined it as an existing diagnosis of substance use in the patient’s chart based on the patient’s past medical history; it was considered separate from a history of alcohol use disorder. The patient population in the area served by the hospital system is medically underserved with a high rate of substance use disorder (with approximately 4% of the total city population receiving substance use disorder treatment over a one-year period),^21^ specifically fentanyl and cocaine, which can result in cardiac arrest through arrhythmias or hypoxia.^21^ ^–^ ^23^

The primary outcome was neurologically intact hospital discharge defined by a modified Rankin scale ≤3, which has been used in previous cardiac arrest literature.^24^ The primary outcome was stratified by total number of doses of epinephrine to evaluate for effect of escalating doses. We considered one dose of epinephrine to be 1 mg administered via an intravenous push, the standard dose recommended by ACLS for cardiac arrest. Secondary outcomes included the age of patients receiving epinephrine doses, the number of epinephrine doses administered by EMS vs the ED, and the time to first epinephrine dose.

Patients were stratified by number of epinephrine doses into three groups: 1–3 doses, 4–6 doses, and ≥7 doses. Other studies used smaller groups (1 dose, 2–5 doses, >5 doses), but we found that our grouping resulted in three groups of similar size, allowing for a more balanced analysis.^6^ The choice of groups was also determined by an estimate of resuscitation time based on administration of a single epinephrine dose every 3–5 minutes on average. The 1–3 dose group implies at least 10 minutes of resuscitation, 4–6 dose group 20 minutes of resuscitation, and ≥7 dose group above 20 minutes of resuscitation. We chose 20 minutes as our upper cutoff since, as mentioned earlier, this represents the time after which the European Resuscitation Council recommends terminating resuscitative efforts.^8^

### Data Collection

We collected data via manual abstraction. The principal investigators (ZB, TS) met to create a standardized method in which data would be abstracted and then held a one-hour session to train the rest of the research team. Each member of the research team (ZB, DD, KD) would collect an equal proportion of data of which 10% was reviewed by a separate investigator (TS) for inter-reviewer reliability and accuracy, and any corrections were made on an ongoing basis. The research team was not blinded to the a priori hypothesis.

We conducted statistical analysis using SPSS version 28 (IBM Corporation, Armonk, NY), using a *P*-value <0.05 for significance. We first tested for normality with a Shapiro-Wilk test. Non-normal data, such as the grouping of patients by epinephrine dosing and age, was analyzed with either a Mann-Whitney *U* test (two groups) or Kruskal-Wallis testing (greater than two groups). Normal data such as demographics, comorbidities, EMS vs ED administration, in- vs out-of-hospital cardiac arrest, and shockable vs non-shockable rhythm, was analyzed with either the Student *t*-test (two groups) or ANOVA testing (greater than two groups).

## RESULTS

**Figure 2.**
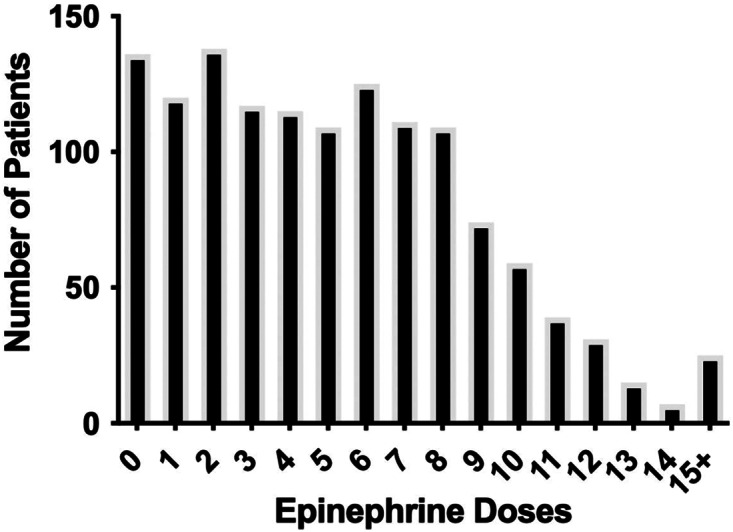
Total epinephrine doses administered.

The study analyzed 1,330 patients, with 136 patients (10.2%) receiving no doses of epinephrine, and 1,194 patients who received at least one dose of epinephrine (Figure 2). The average age of the cohort was 63.3 years old (SD 17.4), and 65.2% male. The total mortality in the study was 1,109 deaths (83.4%) with 653 (58.9%) of those deaths occurring in the ED. The total survival to neurologically intact hospital discharge was 184 patients (13.8%). There were 1,137 (85.5%) out-of-hospital cardiac arrest patients, and 551 patients (41.4%) received at least one defibrillation. Bystander or immediate CPR was initiated in 767 patients (57.6%). Comorbidities were able to be determined for 861 patients (64.7%) from EHR review, while the other patients had no prior visits and no documented comorbidities. Demographics and comorbidities can be found in Table 1, with minor but statistically significant differences between groups. Bystander or immediate CPR was more common if patients received zero or 1–3 doses of epinephrine when compared to higher doses of epinephrine (Table 1).

**Table 1.** Demographics and comorbidities.

	Total cohort (%^a^, n)	0 Doses epinephrine (%^a^, n)	1–3 Doses epinephrine (%^a^, n)	4–6 Doses epinephrine (%^a^, n)	≥7 Doses epinephrine (%^a^, n)
Total patients	1,330	136	375	349	470
Average age	63.3 years	65.0 years	63.8 years	62.3 years	62.9 years
Male*	65.2 (867)	72.8 (99)	57.6 (216)	67.1 (234)	67.7 (318)
Bystander/immediate CPR^*^	57.6 (767)	74.3 (101)	63.5 (238)	53.3 (186)	51.5 (242)
Shockable rhythm^*^	41.9 (557)	60.2 (82)	34.9 (131)	33.5 (117)	48.3 (227)
Non-shockable rhythm^*^	58.1 (773)	39.7 (54)	65.1 (244)	66.5 (232)	51.7 (243)
In-hospital arrest^*^	13.5 (180)	17.6 (24)	19.5 (73)	13.1 (46)	7.9 (37)
Out-of-hospital arrest^*^	88.3 (1,174)	82.4 (112)	80.5 (302)	86.9 (303)	92.1 (433)
Missing comorbidities^*^	35.3 (469)	17.6 (24)	18.1 (68)	23.2 (81)	28.5 (134)
Cardiac risk factors				
HTN^*^	48.2 (641)	59.6 (81)	65.9 (170)	60.2 (129)	59.2 (164)
HLD^*^	29.3 (390)	40.4 (55)	41.0 (106)	34.1 (73)	36.1 (100)
CKD^*^	11.1 (147)	6.6 (9)	15.5 (40)	15.9 (34)	15.5 (43)
DM^*^	25.7 (342)	17.6 (24)	31.0 (80)	33.6 (72)	40.4 (112)
CHF^*^	16.0 (213)	16.2 (22)	24.4 (63)	15.4 (33)	23.5 (65)
MI/CAD^*^	23.2 (308)	37.5 (51)	32.6 (84)	22.9 (49)	28.2 (78)
Lung disease^*^	18.6 (248)	19.9 (27)	25.2 (65)	18.7 (40)	27.8 (77)
Substance use^*^	14.9 (198)	8.8 (12)	20.9 (54)	25.2 (54)	22.0 (61)

^*^ Denotes statistically significant (*P* < 0.05) group difference using an ANOVA test; ^a^Percentages calculated based on total patients in each group.

*CPR*, cardiopulmonary resuscitation; *HTN*, hypertension; *HLD*, hyperlipidemia; *DM*, diabetes mellitus; *CHF*, congestive heart failure; *MI/CAD*, myocardial infarction/coronary artery disease.

A total of 136 patients received zero epinephrine doses, with 89 (65.4%) surviving to a neurologically intact hospital discharge. At least one defibrillation was administered to 63.2% of the zero epinephrine patients, compared to 39.8% of patients who received at least one dose of epinephrine. A resuscitation time was able to be calculated for 91 patients with zero epinephrine doses (66.9%), with a mean resuscitation time of 8.2 minutes and median of 5 minutes.

A total of 1,194 patients received at least one dose of epinephrine. They received an average of 5.8 doses epinephrine (SD 3.6). One hundred and fifty-six patients (13.0%) were in-hospital cardiac arrests. A total of 91 patients (7.6%) survived to a neurologically intact hospital discharge. Average resuscitation time was 33.4 minutes for this cohort. A resuscitation time could be calculated for 1,007 patients (84.3%), with the other patients having no clear prehospital resuscitation time communicated. Average resuscitation time for the 1–3 dose group was 14.8 minutes (min) (median 10 min), for the 4–6 dose group 32.2 min (median 30 min), and for the ≥ 7 doses group 48.5 min (median 50 min).

**Figure 3.**
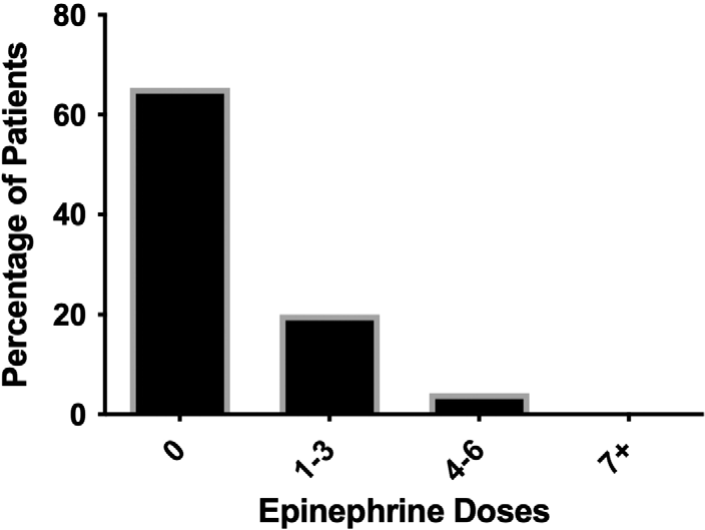
Percentage of patients receiving epinephrine who survived to neurologically intact hospital discharge.

The primary outcome of neurologically intact hospital discharge (Figure 3) was found in 89 (65.4%) patients who received zero doses of epinephrine, 75 (20.0%) patients in the 1–3 dose epinephrine group, 15 (4.3%) in the 4–6 dose group, and one (0.2%) in the ≥7 doses group (*P* < 0.001). Patients who did achieve ROSC had an earlier likelihood for mortality with increasing doses of epinephrine (Supplemental Figure 1).

When compared to the 1–3 dose group, the odds of surviving to hospital admission decreased as the number of epinephrine doses increased for both the 4–6 dose group (odds ratio [OR] 0.58. 95% confidence interval [CI] 0.46–0.74; *P* < 0.001)) and the ≥7 doses group (OR 0.27, 95% CI 0.21–0.35; *P* < 0.001)), as well as the odds for survival to neurologically intact hospital discharge (OR 0.26, 95% CI 0.15–0.47; *P* < 0.001) and (OR 0.01, 95% CI 0.002–0.08; *P* < 0.001) respectively.

The single patient in the cohort who survived to neurologically intact discharge with ≥7 doses of epinephrine was a 63-year-old female who had a shockable rhythm. She received 13 doses of epinephrine and 15 defibrillations, all by EMS, and was ultimately found to have severe coronary artery disease and required a coronary bypass.

### Secondary Outcomes

**Figure 4.**
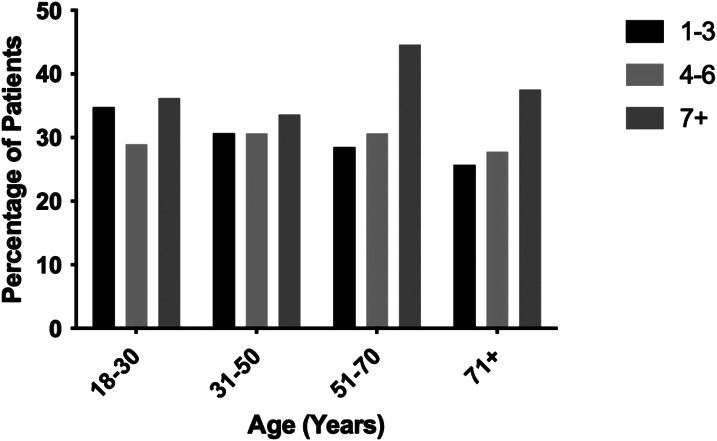
Epinephrine doses distributed by patient age as a percentage of patients within each age group.

**Figure 5.**
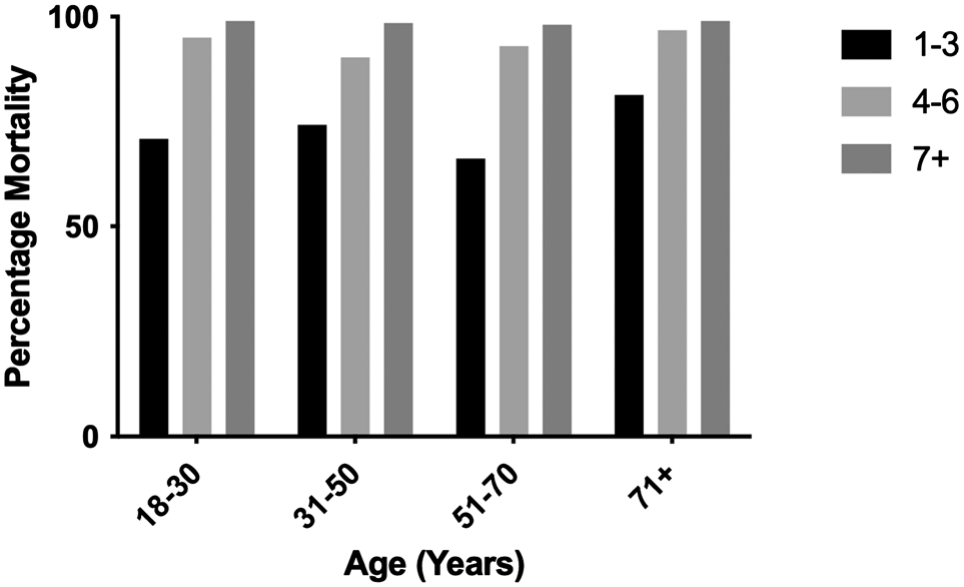
Mortality in patients grouped by age.

Patients who received epinephrine were sub-grouped by age into four cohorts: 18–30 years old; 31–50 years old; 51–70 years old; and ≥71 years old (Figure 4). There was no significant difference between increasing age and increasing doses of epinephrine, as younger patients did not receive ≥7 doses of epinephrine more so than patients ≥71 years old (*P* = 0.80). Patients aged 18-30 years old were also equally likely to receive 1–3 doses of epinephrine when compared to the ≥71 years old group (*P* = 0.11). Overall mortality was also similar between different age groups as well, ranging from 88.4% for the 18–30 group to 92.6% for patients ≥71 years old (*P* = 0.23), and there was no discernible age-based mortality pattern when stratified for epinephrine dosing (Figure 5).

A time to first epinephrine dose was calculated for the 156 in-hospital cardiac arrests who received epinephrine, with those surviving to a neurologically intact hospital discharge (n = 26) receiving their first epinephrine dose on average 1.54 minutes into the resuscitation, compared to those who did not survive to a neurologically intact hospital discharge (n = 130) receiving their first dose at 2.15 minutes, which was statistically significant (*P* = 0.02). Time to first epinephrine dose was not calculated for the out-of-hospital cardiac arrest patients, unfortunately, due to the lack of consistent prehospital data available for retrospective review. Additionally, the survival to neurologically intact hospital discharge for the in-hospital cardiac arrests was 16.0% (n = 26), compared to 6.3% (n = 65) for the out-of-hospital cardiac arrests (*P* < 0.001).

Patients who received epinephrine and had a shockable rhythm at any time during their arrest (n = 475) received an average of 6.25 doses of epinephrine, compared to 5.49 doses in patients who never had a shockable rhythm (*P* < 0.001). Survival to neurologically intact hospital discharge in this subgroup was 10.4% (49 patients) with a shockable rhythm and 5.8% (42 patients) with a nonshockable rhythm (*P* < 0.001). A shockable rhythm was also seen more in the zero-dose epinephrine group and the ≥7 dose group, and a non-shockable rhythm was seen more in groups receiving epinephrine compared to those who did not receive epinephrine (Table 1).

There were also differences between number of epinephrine doses administered by EMS compared to the ED. Of the out-of-hospital cardiac arrest patients who received epinephrine, the ED was more likely to administer zero doses of epinephrine (34.9%) than EMS (5.9%) (*P* < 0.001). Patients who suffered an in-hospital arrest were more likely to receive zero doses or 1–3 doses, while patients who suffered an out-of-hospital cardiac arrest were more likely to receive ≥7 doses of epinephrine (Table 1). Of 1,036 out-of-hospital cardiac arrests, 162 (15.6%) received ≥7 epinephrine doses by EMS. Of the 158 in-hospital cardiac arrests, 37 patients (23.4%) received ≥7 doses of epinephrine in the ED. A total of 980 patients (82.1%) received their first dose of epinephrine from EMS, with 60 (6.1%) surviving to neurologically intact hospital discharge, compared to 214 patients who received their first dose of epinephrine from the ED, with 31 (14.5%) surviving to a neurologically intact hospital discharge (*P* < 0.001). Sixty-four patients who received their first dose of epinephrine in the ED were defined as out-of-hospital cardiac arrests, with nine (14.0%) patients achieving ROSC by EMS and had subsequent loss of pulses in the ED. Nine (14.0%) of this patient subset survived to a neurologically intact hospital discharge.

## DISCUSSION

Our results show a significant decrease in patients with neurologically intact discharges as the number of epinephrine doses increase. In addition, receiving ≥7 doses of epinephrine was associated with a very low probability of achieving ROSC (1/470, 0.2%). This study supports the findings of Jouffroy et al that ≥7 doses of epinephrine decreased the chances of obtaining ROSC.^25^ The secondary finding that patients with a shockable rhythm or in-hospital cardiac arrest received more epinephrine doses than their counterparts suggests that physicians may continue giving epinephrine doses due to the known higher survivability of these patient subgroups in prior studies, which our findings corroborated.^26^ ^,^ ^27^ We did not find, however, that younger patients received more epinephrine doses, regardless of how much total epinephrine was given. Patients older than 70 received ≥7 doses of epinephrine in similar proportions to patients 18–30. Mortality was also similar between age groups within each subgroup of epinephrine dosing. This finding, to our knowledge, does not appear in the current literature on epinephrine use in cardiac arrest and suggests that physicians are not taking into consideration patient age during resuscitation, or that the patient’s age is less of a factor to cease resuscitation. While the study was not specifically designed to compare patient age and number of epinephrine doses, this novel finding is hypothesis-generating and should be studied in a prospective fashion.

Multiple studies address the futility of increasing the number of epinephrine doses in cardiac arrest, finding that increased epinephrine doses was associated with increased mortality for patients.^6^ ^,^ ^10^ ^,^ ^12^ ^,^ ^28^ Our study results agreed with those findings and increase their generalizability to an overall ED population, in that we conducted a secondary analysis of patients by age to show that younger patients did not skew our results and that we included both in-hospital and out-of-hospital cardiac arrests, shockable and non-shockable rhythms, and EMS to ED administration of epinephrine, unlike previous research.

A study by Grunau et al did show survival with neurologically intact discharge in a small number of patients who received >7 doses of epinephrine, but they did not elaborate on these patients as it was not a study outcome.^29^ Shi et al showed neurologically intact survival in 23.6% (17/72) of patients receiving ≥7 epinephrine doses, with 12 being shockable rhythms, 14 receiving bystander CPR, and all but one patient receiving targeted temperature management (TTM).^12^ This survival rate is much higher than reported in previous literature on cardiac arrest patients receiving large doses of epinephrine and was neither an intended nor a reported final outcome of the study. Because there is some evidence that patients receiving ≥7 epinephrine doses may survive neurologically intact, there may be a subgroup of patients who would benefit from >6 doses of epinephrine, and the true point of epinephrine futility may lie at a higher dose.

While our data suggests the likelihood of neurologically intact hospital discharge decreases as epinephrine doses increase, there are confounders to address which of them affect the conclusions that can be drawn from our data. The average resuscitation time for each group increased with the increasing number of epinephrine doses. Although resuscitation time is directly and inexorably linked with increasing epinephrine use, our study also aimed to evaluate use of epinephrine during cardiac arrest, which is a factor that clinicians have direct control over. The significant difference for the time to first epinephrine for the in-hospital cardiac arrests is also a confounder, as previous studies have shown that a delay to first epinephrine increases mortality and results in worse neurologic outcomes.^30^ We had only 14.5% of patients sustain an in-hospital cardiac arrest, which limited the statistical power of this finding, and only 25% of the in-hospital cardiac arrest patients received ≥7 doses of epinephrine compared to 40% of the out-of-hospital cohort.

Additionally, we found that 64 patients who were classified as out-of-hospital cardiac arrests, who did not receive EMS epinephrine but received epinephrine in the ED, still survived to a neurologically intact hospital discharge at a high rate (14.0%). This finding was either due to a responding EMS crew with emergency medical technicians who were unable to administer medications, an inability to obtain intravenous or intraosseous access, or ROSC being achieved prior to administration of epinephrine with a subsequent cardiac arrest in the ED. We did not include these 64 patients in our time to first epinephrine calculations as no initial time point was available to calculate forward from. If this data was available, it may have changed the association between decreased time to first epinephrine and increased neurologically intact hospital discharge as we would expect the time to first epinephrine to be longer in this population.

We feel that despite these confounders, our data is accurate in showing an association between decreased neurologically intact hospital discharge as the number of epinephrine doses increases. Additionally, there are multiple factors that go into clinicians deciding when to cease resuscitation such as patient age, potential cause of cardiac arrest, initial cardiac rhythm and any defibrillations received. Our novel secondary outcomes attempt to describe these factors to give further insight into which patients receive more epinephrine doses.

Our data showing that ≥7 doses of epinephrine can be considered futile may have an impact not only on resources and duration of ACLS but may also be another tool for guiding goals-of-care conversations with families. If patients received ≥7 doses of epinephrine and achieved ROSC, physicians could better set expectations regarding the exceedingly poor prognosis of these patients. It may also be useful guiding these discussions in patients actively undergoing CPR, relaying the poor outcomes and helping families make decisions on continued resuscitation vs termination of efforts.

## LIMITATIONS

The major limitation of this study was the absence of time to first epinephrine, which was due to inconsistent EMS data, as the majority of our cases were out-of-hospital cardiac arrests. This is also due to the retrospective nature of the study, which comes with its own limitations. It would be difficult to perform a prospective trial due to concern regarding the standard of care, and thus it is likely not feasible at this time to evaluate the number, and effect, of epinephrine doses a patient should receive.^31^ Additionally, missing data limited our sample size, as we eliminated any patients with missing epinephrine data from the study. We did not have comorbidity data on approximately 37% of the cohort due to patients not having prior ED visits within the hospital system where this data could have been collected.

We do not have access to autopsy or post-mortem health information in our EHR, and so we were unable to effectively screen all patients for comorbidities. There were statistically significant differences between groups for patient comorbidities, but we believe the patient comorbidities may not have had a significant role in the amount of epinephrine patients were administered as they may not have been known to EMS and emergency clinicians at the time. Also, information abstracted from chart review can be inaccurate, but we reduced the possibility of inaccurate data by reviewing both physician and nursing notes along with the code documentation sheet to reconcile all differences.

Our cohort’s neurologically intact survival of 13.8% was higher than other reported studies, which we suspect may have been due to us including both in-hospital and out-of-hospital cardiac arrest. We also included patient who received zero doses of epinephrine, who overall had a 65.4% rate of neurologically intact discharge. Finally, we were limited in our assessment of ACLS time as many charts were missing a definitive time of resuscitation, especially for out-of-hospital cardiac arrests, which made up the majority of our dataset. We often found documentation of exact total prehospital ACLS time by emergency physicians often stopped at one hour, which likely led to an underestimation of the duration of resuscitation. When possible, we gathered as much data as possible using the documentation that was available to us via our ED’s EHR.

## CONCLUSION

This study of epinephrine dosing in cardiac arrest suggests that ≥7 doses of epinephrine is almost always futile within our institution. These results will need to be prospectively studied at other institutions and in different patient populations to confirm their accuracy. Additionally, our data suggests that patients with favorable survival odds from a shockable rhythm or an in-hospital cardiac arrest receive more epinephrine doses, although the difference is small and may not be clinically relevant. In contrast, however, younger adults did not receive more epinephrine doses than older adults, a finding that should be studied further. This study highlights valuable concepts that physicians should be aware of when considering the futility or benefit of continued resuscitation efforts.

## Supplementary Information





## References

[1] Panchal AR Bartos JA Cabañas JG et al. Part 3: Adult Basic and Advanced Life Support: 2020 American Heart Association Guidelines for Cardiopulmonary Resuscitation and Emergency Cardiovascular Care. Circulation. 2020;142(16_suppl_2):S366–468. 33081529 10.1161/CIR.0000000000000916

[2] Velissaris D Karamouzos V Pierrakos C et al. Use of sodium bicarbonate in cardiac arrest: current guidelines and literature review. J Clin Med Res. 2016;8(4):277–83. 26985247 10.14740/jocmr2456w PMC4780490

[3] Kette F Ghuman J Parr M. Calcium administration during cardiac arrest: a systematic review. Eur J Emerg Med. Apr 2013;20(2):72–8. 22990036 10.1097/MEJ.0b013e328358e336

[4] Olasveengen TM Wik L Sunde K et al. Outcome when adrenaline (epinephrine) was actually given vs. not given - post hoc analysis of a randomized clinical trial. Resuscitation. 2012;83(3):327–32. 22115931 10.1016/j.resuscitation.2011.11.011

[5] Hagihara A Hasegawa M Abe T et al. Prehospital epinephrine use and survival among patients with out-of-hospital cardiac arrest. JAMA. 2012;307(11):1161–8. 22436956 10.1001/jama.2012.294

[6] Dumas F Bougouin W Geri G et al. Is epinephrine during cardiac arrest associated with worse outcomes in resuscitated patients? J Am Coll Cardiol. 2014;64(22):2360–7. 25465423 10.1016/j.jacc.2014.09.036

[7] Jacobs IG Finn JC Jelinek GA et al. Effect of adrenaline on survival in out-of-hospital cardiac arrest: a randomised double-blind placebo-controlled trial. Resuscitation. 2011;82(9):1138–43. 21745533 10.1016/j.resuscitation.2011.06.029

[8] Baskett PJ Nolan JP Handley A et al. European Resuscitation Council Guidelines for Resuscitation 2005. Section 9. Principles of training in resuscitation. Resuscitation. 2005;67(Suppl 1):S181–9. 16321713 10.1016/j.resuscitation.2005.10.006

[9] Morrison LJ Kierzek G Diekema DS et al. Part 3: ethics: 2010 American Heart Association Guidelines for Cardiopulmonary Resuscitation and Emergency Cardiovascular Care. Circulation. 2010;122(18 Suppl 3):S665–75. 20956219 10.1161/CIRCULATIONAHA.110.970905

[10] Arrich J Sterz F Herkner H et al. Total epinephrine dose during asystole and pulseless electrical activity cardiac arrests is associated with unfavourable functional outcome and increased in-hospital mortality. Resuscitation. 2012;83(3):333–7. 22079948 10.1016/j.resuscitation.2011.10.027

[11] Goto Y Maeda T Goto Y. Effects of prehospital epinephrine during out-of-hospital cardiac arrest with initial non-shockable rhythm: an observational cohort study. Crit Care (London, England). 2013;17(5):R188. 10.1186/cc12872 PMC3840562 24004456

[12] Shi X Yu J Pan Q et al. Impact of total epinephrine dose on long term neurological outcome for cardiac arrest patients: a cohort study. Front Pharmacol. 2021:12:580234. 34122055 10.3389/fphar.2021.580234 PMC8193671

[13] de Oliveira FC Feitosa-Filho GS Ritt LE. Use of beta-blockers for the treatment of cardiac arrest due to ventricular fibrillation/pulseless ventricular tachycardia: a systematic review. Resuscitation. 2012;83(6):674–83. 22306254 10.1016/j.resuscitation.2012.01.025

[14] Kagawa E Inoue I Kawagoe T et al. Assessment of outcomes and differences between in- and out-of-hospital cardiac arrest patients treated with cardiopulmonary resuscitation using extracorporeal life support. Resuscitation. 2010;81(8):968–73. 20627526 10.1016/j.resuscitation.2010.03.037

[15] Avalli L Maggioni E Formica F et al. Favourable survival of in-hospital compared to out-of-hospital refractory cardiac arrest patients treated with extracorporeal membrane oxygenation: an Italian tertiary care centre experience. Resuscitation. 2012;83(5):579–83. 22056265 10.1016/j.resuscitation.2011.10.013

[16] Ristagno G Sun S Tang W et al. Effects of epinephrine and vasopressin on cerebral microcirculatory flows during and after cardiopulmonary resuscitation. Crit Care Med. 2007;35(9):2145–9. 17855828 10.1097/01.ccm.0000280427.76175.d2

[17] Rivers EP Wortsman J Rady MY et al. The effect of the total cumulative epinephrine dose administered during human CPR on hemodynamic, oxygen transport, and utilization variables in the postresuscitation period. Chest. 1994;106(5):1499–507. 7956410 10.1378/chest.106.5.1499

[18] Fernando SM Mathew R Sadeghirad B et al. Epinephrine in out-of-hospital cardiac arrest: a network meta-analysis and subgroup analyses of shockable and nonshockable rhythms. Chest. 2023;164(2):381–93. 36736487 10.1016/j.chest.2023.01.033

[19] Evans E Swanson MB Mohr N et al. Epinephrine before defibrillation in patients with shockable in-hospital cardiac arrest: propensity matched analysis. BMJ. 2021;375:e066534. 34759038 10.1136/bmj-2021-066534 PMC8579224

[20] Merchant RM Topjian AA Panchal AR et al. Part 1: Executive Summary: 2020 American Heart Association Guidelines for Cardiopulmonary Resuscitation and Emergency Cardiovascular Care. Circulation. 2020;142(16_suppl_2):S337–57. 33081530 10.1161/CIR.0000000000000918

[21] Navarretta N. Annual Statistics Report SFY 2019. Connecticut Department of Mental Health and Addiction Services. 2019.

[22] Kim ST Park T. Acute and chronic effects of cocaine on cardiovascular health. Int J Mol Sci. 2019;20(3):584. 30700023 10.3390/ijms20030584 PMC6387265

[23] Paredes VL Rea TD Eisenberg MS et al. Out-of-hospital care of critical drug overdoses involving cardiac arrest. Acad Emerg Med. 2004;11(1):71–4. 14709431 10.1197/j.aem.2003.08.014

[24] Banks JL Marotta CA. Outcomes validity and reliability of the modified Rankin scale: implications for stroke clinical trials. Stroke. 2007;38(3):1091–6. 17272767 10.1161/01.STR.0000258355.23810.c6

[25] Jouffroy R Saade A Alexandre P et al. Epinephrine administration in non-shockable out-of-hospital cardiac arrest. Am J Emerg Med. 2019;37(3):387–90. 29857945 10.1016/j.ajem.2018.05.055

[26] Bonnin MJ Pepe PE Clark PS Jr. Survival in the elderly after out-of-hospital cardiac arrest. Crit Care Med. 1993;21(11):1645–51. 8222679 10.1097/00003246-199311000-00012

[27] Grunau B Reynolds JC Scheuermeyer FX et al. Comparing the prognosis of those with initial shockable and non-shockable rhythms with increasing durations of CPR: informing minimum durations of resuscitation. Resuscitation. 2016;101:50–6. 26851705 10.1016/j.resuscitation.2016.01.021

[28] Fukuda T Ohashi-Fukuda N Matsubara T et al. Effect of prehospital epinephrine on out-of-hospital cardiac arrest: a report from the national out-of-hospital cardiac arrest data registry in Japan, 2011-2012. Eur J Clin Pharmacol. 2016;72(10):1255–64. 27411936 10.1007/s00228-016-2093-2

[29] Grunau B Kawano T Scheuermeyer FX et al. The association of the average epinephrine dosing interval and survival with favorable neurologic status at hospital discharge in out-of-hospital cardiac arrest. Ann Emerg Med. 2019;74(6):797–806. 31248676 10.1016/j.annemergmed.2019.04.031

[30] Hansen M Schmicker RH Newgard CD et al. Time to epinephrine administration and survival from nonshockable out-of-hospital cardiac arrest among children and adults. Circulation. 2018;137(19):2032–40. 29511001 10.1161/CIRCULATIONAHA.117.033067 PMC5940513

[31] Jung J Rice J Bord S. Rethinking the role of epinephrine in cardiac arrest: the PARAMEDIC2 trial. Ann Transl Med. 2018;6(Suppl 2):S129. 30740450 10.21037/atm.2018.12.31 PMC6330609

