# Supplementary material for: Epinephrine in Cardiac Arrest: Identifying a Potential Limit for Resuscitation
Source: West J Emerg Med. 2023 Nov 8;24(6):1025–33. doi: 10.5811/westjem.60840 (PMC10754196; doi:10.5811/westjem.60840)
Supplement: Supplementary file 1 [file wjem-24-1025-s001.docx]

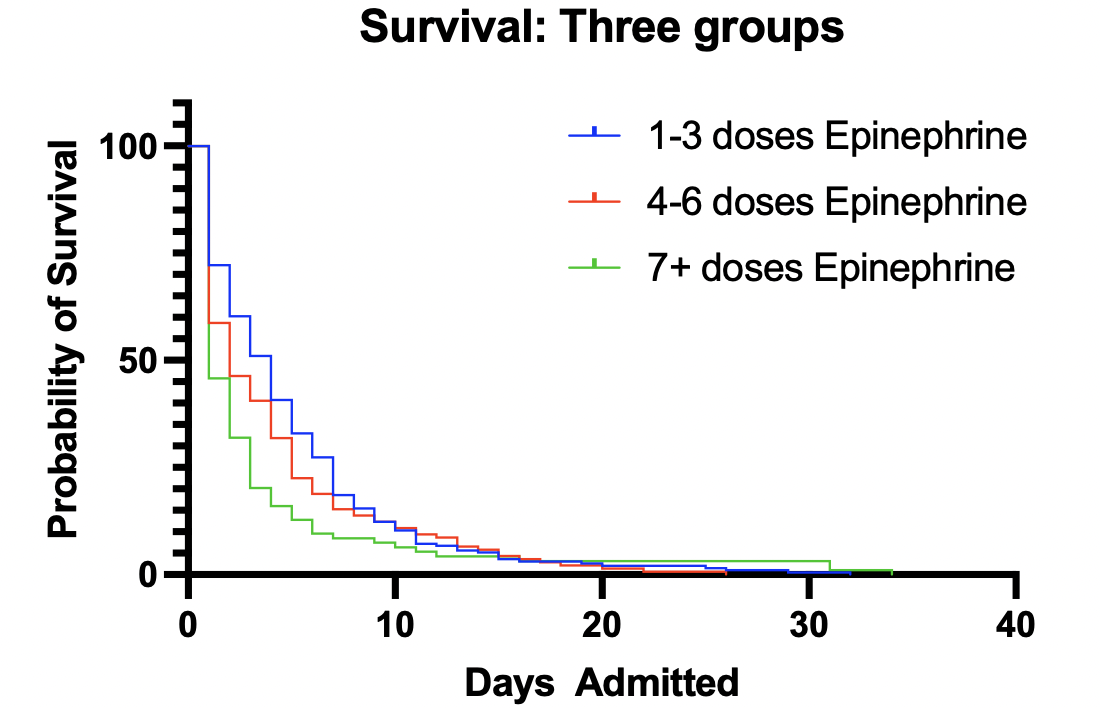


**Supplemental Figure 1.** Kaplan Meier plot of epinephrine doses and mortality in patients with in-hospital mortality.
